# Hypertension in Guatemala’s Public Primary Care System: A Needs Assessment Using the Health System Building Blocks Framework

**DOI:** 10.1186/s12913-021-06889-0

**Published:** 2021-09-03

**Authors:** Meredith P. Fort, William Mundo, Alejandra Paniagua-Avila, Sayra Cardona, Juan Carlos Figueroa, Diego Hernández-Galdamez, Kristyne Mansilla, Ana Peralta-García, Dina Roche, Eduardo Alberto Palacios, Russell E. Glasgow, Pablo Gulayin, Vilma Irazola, Jiang He, Manuel Ramirez-Zea

**Affiliations:** 1 Department of Health Systems, Management and Policy, Centers for American Indian and Alaska Native Health, Colorado School of Public Health, Anschutz Medical Campus, Aurora, CO USA; 2 Adult and Child Consortium for Health Outcomes Research and Delivery Science, University of Colorado School of Medicine, Anschutz Medical Campus, Aurora, CO USA; 3 INCAP Research Center for the Prevention of Chronic Diseases (CIIPEC), Institute of Nutrition of Central America and Panama (INCAP), Guatemala City, Guatemala; 4 University of Colorado School of Medicine, Anschutz Medical Campus, Aurora, CO USA; 5 Department of Epidemiology, Mailman School of Public Health, Columbia University, New York City, NY USA; 6 Ministry of Health and Social Welfare, Guatemala City, Guatemala; 7 South American Center of Excellence for Cardiovascular Health, Department of Research in Chronic Diseases, Institute for Clinical Effectiveness and Health Policy (IECS), Buenos Aires, Argentina; 8 Tulane University School of Public Health and Tropical Medicine, New Orleans, LA USA; 9 Tulane University Translation Science Institute, New Orleans, LA USA

**Keywords:** Cardiovascular disease, Guatemala, Health system building blocks, Hypertension, Implementation science, Low- and middle-income countries, Multicomponent program, Non-communicable diseases, Primary care

## Abstract

**Background:**

Uncontrolled hypertension represents a substantial and growing burden in Guatemala and other low and middle-income countries. As a part of the formative phase of an implementation research study, we conducted a needs assessment to define short- and long-term needs and opportunities for hypertension services within the public health system.

**Methods:**

We conducted a multi-method, multi-level assessment of needs related to hypertension within Guatemala’s public system using the World Health Organization’s health system building blocks framework. We conducted semi-structured interviews with stakeholders at national (n = 17), departmental (n = 7), district (n = 25), and community (n = 30) levels and focus groups with patients (3) and frontline auxiliary nurses (3). We visited and captured data about infrastructure, accessibility, human resources, reporting, medications and supplies at 124 health posts and 53 health centers in five departments of Guatemala. We conducted a thematic analysis of transcribed interviews and focus group discussions supported by matrix analysis. We summarized quantitative data observed during visits to health posts and centers.

**Results:**

Major challenges for hypertension service delivery included: gaps in infrastructure, insufficient staffing and high turnover, limited training, inconsistent supply of medications, lack of reporting, low prioritization of hypertension, and a low level of funding in the public health system overall. Key opportunities included: prior experience caring for patients with chronic conditions, eagerness from providers to learn, and interest from patients to be involved in managing their health. The 5 departments differ in population served per health facility, accessibility, and staffing. All but 7 health posts had basic infrastructure in place. Enalapril was available in 74% of health posts whereas hydrochlorothiazide was available in only 1 of the 124 health posts. With the exception of one department, over 90% of health posts had a blood pressure monitor.

**Conclusions:**

This multi-level multi-method needs assessment using the building blocks framework highlights contextual factors in Guatemala’s public health system that have been important in informing the implementation of a hypertension control trial. Long-term needs that are not addressed within the scope of this study will be important to address to enable sustained implementation and scale-up of the hypertension control approach.

**Supplementary Information:**

The online version contains supplementary material available at 10.1186/s12913-021-06889-0.

## Background

Uncontrolled hypertension represents a substantial and growing health burden in Guatemala and many other low- and middle-income countries (LMICs). According to the Global Burden of Disease, high systolic blood pressure is the leading risk factor for mortality and disability-adjusted life-years [1]. Many health systems, especially in LMICs, lack developed programs that address non-communicable diseases (NCD). An analysis of hypertension control in 44 LMICs found that only 10% of people with hypertension achieved blood pressure control [2].

There is recognition that health systems in LMIC contexts need support to improve their capacity to deliver interventions focused on the management of NCDs [3]. The World Health Organization’s (WHO) health system building blocks framework can help define priority needs within different components of the healthcare system [4]. The six building blocks are: service delivery, human resources, medications and technologies, health information systems, financing, and governance and leadership. The building blocks provide a way of viewing the system in its entirety rather than just one component, although it does not capture interactions between levels [5].

Prior to initiating the trial phase of an implementation research study aimed at improving hypertension control in rural Guatemala [6], we conducted a multi methods needs assessment of the health system building blocks as they relate to hypertension prevention and care. This study is part of the HyTREC/TREIN, NHLBI-funded consortium of projects that aims to increase capacity in LMICs to design, implement, and assess interventions that address the cardiovascular disease burden globally and to improve the global health community’s understanding of the barriers and opportunities specific to LMICs and the strategies best suited for low-resource health care settings [7]. The study is led by the Institute of Nutrition of Central America and Panama (INCAP), a regional health and nutrition research institute based in Guatemala City, in coordination with the Guatemalan Ministry of Health and Social Welfare and researchers at the Insitute for Clinical Effectiveness and Health Policy, Tulane University and the Colorado School of Public Health.

For the hypertension control study in Guatemala, we are interested in the sustainability potential and the extent that the intervention and implementation strategies fit the context, in addition to the effectiveness of the intervention. Our study is a type 2 hybrid effectiveness-implementation study [8] in which we are employing the expanded RE-AIM (Reach, Effectiveness, Adoption, Implementation, Maintenance) [9, 10] - PRISM (the Practical, Robust and Sustainability Model) [11] model. In implementation science research it is important to understand different levels of contextual factors and perspectives within a system in order to prepare relevant multi-level strategies [12]. As such, this needs assessment captures the perspectives at different levels within the Guatemalan public primary care system and the community.

The Guatemalan Ministry of Health and Social Welfare (MoH) is the primary agency in the country’s health system and is responsible for its oversight and service delivery to the majority of the population [13]. The MoH is administratively organized with a central level in Guatemala City, Health Areas within each of the departments, and Health Districts which represent municipal administration. The service delivery system is organized into primary, secondary and tertiary levels of care; health posts are at the primary, community level and refer to health centers which in turn refer to regional and national hospitals [14]. Health posts are staffed by auxiliary nurses and health centers are staffed by physicians, professional and auxiliary nurses, as well as psychologists and social workers, in some cases. Guatemala’s health system faces many of the same challenges seen in other LMIC settings incluing fragmentation and high out-of-pocket costs – which represent more than 50% of total health costs [13].

The purpose of this paper is to assess the primary needs of the public sector health system in Guatemala with respect to hypertension prevention and care. The secondary aim is to present the current capacity within the health posts and centers where the trial intervention is being implemented (2019-2021) [15]. The needs assessment serves as a baseline assessment of the public health care system with respsect to hypertension prevention, detection and treatment within the public primary care system in Guatemala. This article points to key needs and areas to be strengthened – in the short and long term - and we share recommendations and implications for the prevention and care of hypertension and other NCDs within a health system strengthening perspective, and consider how to operationalize the ongoing capture and analysis of health system contextual data within implementation research studies.

## Methods

### Data Collection

From February-October 2018 we conducted qualitative and quantitative data gathering at the national, departmental, district, and community levels. The MoH granted approval for the study and facilitated communication and support for data collection. For the first phase of the needs assessment JCF and SC, both trained in social science fields – anthropology and psychology, respectively with previous qualitative research experience, conducted individual interviews with central-level key informants based in Guatemala City. Following this, JCF and SC conducted interviews in two MoH Health Areas (Sololá and Zacapa); these are two of the five Health Areas included in the intervention research study, and are in two distinct regions (the Western highlands and the Eastern part of the country). Within each Health Area, we selected one MoH Health District (La Unión, Zacapa and San Pablo, Sololá) to interview health providers, patients, family members and community members; the Health Districts were selected because they had health posts staffed with two auxiliary nurses, were to be included in the study, and to capture different experiences implementing primary care service delivery models; in the case of Sololá where there were multiple options, San Pablo was selected at random. JCF and SC also conducted focus group discussions (FGDs) with auxiliary nurses and patients. For all interviews and FGDs, JCF and SC introduced themselves as members of the study team hired by INCAP and all participants were given written informed consent. Local translators assisted with interviews conducted in Mayan languages.

We used purposive sampling to identify a similar set of actors at the Health Area, District and community levels. We selected participants within the MoH based on their role and included: Health Area directors, staff familiar with chronic disease programs, and team members responsible for medications. At the Health District level, we interviewed district directors, doctors, nurses, and auxiliary nurses who work at the health center and health post levels; we made sure to interview both male and female health care providers and those located in an urban center and those working in remote settings. At the community level, we interviewed hypertensive patients, family members, and community-level health experts (traditional birth attendants and local practitioners), and community leaders. For patients, we made certain to interview: male and female patients, those living near to and far from health posts/centers, and those who speak Spanish as well as those who speak a Mayan language; patients were at least 40 years of age and were not pregnant at the time of the interview. Potential interview participants were approached via email (MOH staff), phone and face-to-face (patients, community members); for FGDs, staff at health posts and centers reached out to participants to invite them. Some participants declined to participate stating that they did not have time and others were not possible to locate them at the time of the interview or FGDs that they agreed to. Interviews and FGDs were conducted in participants’ homes, community spaces, and MOH facilities and administrative buildings. In some cases, family members who were not participants were present. Interviews continued until all purposive categories were filled and thematic saturation was later confirmed during analysis. The interview and FGD guides, for which questions were tested for understandability prior to field interviews, are presented in Supplementary file 1. Interviews lasted from 10 to 90 min and FGDs were 60-90 min and repeat interviews were not conducted. Interviews and FGDs were recorded and subsequently transcribed.

The final phase of the needs assessment included site visits to health centers and posts considered for participation in the trial in 5 departments (Chiquimula, Baja Verapaz, Zacapa, Sololá, and Huehuetenango). During the site visits, AP-A and KM (medical doctors with public health training) and other members of the project team and data collection assistants filled out electronic data collection forms to capture existing capacity for aspects of the building blocks relevant to the hypertension control intervention. The forms were defined by the project and included assessment of: types of access to the post/center, number and types of health care providers, infrastructure, population served, quantity and types of hypertension medications, availability of blood pressure monitor(s), and clinical forms and educational metarials in use.

**Fig. 1:**
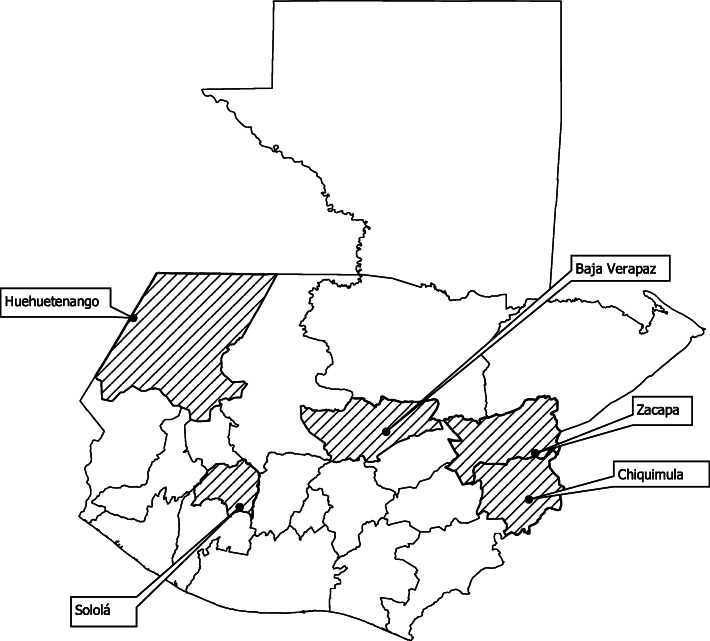
Map of five departments included in the hypertension control study in Guatemala, prepared for the study

After reviewing potential sites with the MoH, we visited 124 health posts in 53 health districts from which we selected 71 health posts and 36 health districts for inclusion in the intervention study. 18 health districts were randomly assigned to the intervention group and 18 to the control group across the 5 departments (as shown in Fig. 1**).**

### Data Analysis

#### Qualitative component (February-August 2018)

All interviews and FGDs were transcribed and uploaded to NVivo v11. We used a combination of grounded theory and a framework analysis applying the health system building blocks framework. JCF and SC coded transcripts together manually based on the interview guide and added codes that emerged from the data. This defined a first codebook that was then transferred to NVivo. JCF and SC came to agreement on coding through discussion and shared the coding process with MPF and AP-A to continually review codes for consistency and resolve questions. The first coding cycle focused on the six building blocks (service delivery, human resources, information systems, medications, financing, and governance). A second coding cycle focused on implications for the hypertension control trial, and short and long-term needs. The coding tree is available as Supplementary file 2. For this analysis, we compared the identified needs to the proposed intervention components and strategies and organized those that were within the scope of the intervention to those that would require additional investment outside of the study.

MPF and WM, public health trained researchers with qualitative methods training and experience, used a matrix analysis to compare responses to questions about the six building blocks by type of actor and levels within the health care system [16]. We also identified cross-cutting themes that were not linked to one specific building block. We then reviewed the transcripts again to confirm our findings and to identify illustrative quotes. MPF and WM translated the illustrative quotes into English and edited only for ease of reading. Summary reports of findings were shared with collaborators within the MoH.

#### Site visits to health centers and posts (August-October 2018)

Quantitative data collected during site visits were entered into a web-based database developed using REDCap [17]. AP-A, DH-G, KM, AP-G, DR, and MPF conducted a descriptive analysis of the components relevant to the building blocks focusing on service delivery and infrastructure, medications and technologies, and health care workers.

## Results

Table 1 summarizes the type of actors who participated in interviews and focus group discussions**.** In total, we conducted 83 interviews and 6 focus group discussions. 17 interviews were conducted at the central level in Guatemala City with key informants. We conducted interviews with MoH providers and staff at different levels in the healthcare system: 7 at the department-level and 25 at the district and health post levels. 12 community leaders and 19 patients and family members were interviewed. In addition, we conducted 3 focus group discussions with auxiliary nurses and 3 with patients.

**Table 1:** Interview and focus group discussion participants from a hypertension needs assessment in Guatemala’s public primary care system

Type of actor	Location	Interviews	Focus group discussions
Central level key informant	Guatemala City	17	
Department level Ministry of Health staff	Zacapa	3	
	Sololá	4	
District level Ministry of Health staff and local level providers	Zacapa	13	2 (11 & 6 auxiliary nurses)
	Sololá	12	1 (9 auxiliary nurses)
Community leaders	Zacapa	6	
	Sololá	6	
Patients and family members	Zacapa	10	2 (9 & 4 patients)
	Sololá	9	1 (8 patients)
**Total**		**83**	**6**

Table 2 presents challenges and opportunities for hypertension prevention care within the public primary health care system based on the perspectives of stakeholders at different levels of the health system. In the table, we organized the challenges and opportunities by the six building blocks.

**Table 2:** Challenges and opportunities for hypertension services captured by stakeholder level and health system building block

Health system building block	Level of actor		
	Central level (Guatemala City)	Providers (Department & district levels)	Community level (patients, family members and community leaders)
***Service delivery***	- Insufficient coverage and limited service unit infrastructure - Lack of laboratory capacity	- Different models of service delivery implemented at the primary care level - Copies of treatment guidelines often not available	Patients are uncertain about how to manage their disease and when they need to seek care
***Human resources***	- Need for enhanced teams at the primary care level - High staff turnover	- Limited opportunities for professional growth; short-term contracts - Limited training on hypertension and in-service “cascade” trainings miss frontline providers	Limited consultation with community members (leaders, traditional healers) in some settings
***Information systems***	- Lack of a standardized clinical encounter form - Undercounting of people with hypertension in clinical encounters	Some but not all sites have lists of patients with hypertension & other chronic conditions	Patients do not keep track of their blood pressure
***Medications and technologies***	Hypertension medications are not consistently available (if available, primarily Enalapril)	- Often lacking medications for hypertension (if available, primarily Enalapril) - Lack of blood pressure monitors in some settings	Patients use traditional medicine in addition to pharmaceutical medications
***Financing***	Public health system is underfunded	Health Areas manage the budgets (not the local, district level)	Patients are unable to afford medications (when there are stock-outs)
***Governance & leadership***	Need for increased capacity for intersectoral collaboration to address chronic diseases	Opportunity for increased coordination between community members and providers	Patients with hypertension at times participate in diabetes clubs; eagerness to become more actively involved in their care

The major challenges identified by stakeholders were: a gap in infrastructure, inequity in coverage between urban and rural areas, insufficient staffing, limited training in hypertension, high staff turnover, lack of blood pressure monitors, limited and inconsistent supply of medications, lack of data capture and reporting in practice, a low level of funding in the public health system, and low prioritization of hypertension and chronic diseases compared to communicable diseases and maternal and child care. Opportunities that were identified to strengthen the response at the community level were: the experience in some settings of implementing a model of service delivery that is organized to combine community and clinic work and to follow-up patients with chronic conditions, an eagerness from healthcare providers to learn more and take on additional work with appropriate support, and an interest from patients to become more involved in managing their hypertension and improving their health. Following are perspectives on needs and opportunities for each of the health system building blocks from stakeholders who are based in different parts of the health system.

### Service delivery

The gap in health care service infrastructure in the country was identified as a major challenge for service delivery: *“There are approximately 2,000 service units, leaving a gap of more than 3,000 service units. So, for the level of detection that we can have if we compare it to the number of service units that we have, it is inadequate. We need to have more service units or create models of care that can work outside of physical infrastructure.”* (Central-level actor).

In addition to the overall gap in health facilities, there is a notable difference in coverage for rural and urban areas with rural areas, which are poorer, having less infrastructure and of lower quality than urban areas: *“Because the problem that we have is that the rural area … it’s a poor service, since it is for the rural area it doesn’t matter that the health unit is made of wood slats, no problem. Poor services for poor people. So we have to change this vision. If I am going to work in a poor area, the units should be much stronger than what is in an urban area.”* (Central-level actor).

There are different implementation strategies or service delivery approaches being implemented at the local level within the public primary care system that may present an opportunity for adding in a hypertension control program. An auxiliary nurse described the model where they work in La Unión, Zacapa which differs from the standard approach to care in the country: *“Right now I respond to everything that comes in to the health post – providing advice, clinical consults, vaccination … that is all a part of my responsibility as the auxiliary nurse who is in-clinic that’s what corresponds to me, right. And my colleague, she takes on activities out in the community … I go out next week … I will change from what I am doing in here to carrying out work in the community.”* A second auxiliary nurse in La Unión, Zacapa explained how the model proactively seeks patients with chronic conditions: ***“****In 2011 we began to work with a new model, and that is when we began to pay attention to diabetics, hypertensive patients, and we began to follow up with them … We gave them an invitation to visit the health post … Maybe there was somebody with hypertension, but he did not show up, so we would go visit him, and then have him come to the health post.”*

Treatment guidelines are not available to all health staff. A central-level actor explained that because the guideline documentation is not given to all providers, it becomes a tool that is inaccessible: “*… because they only give one per service unit, they give it to the Director of the health center, he locks it away so that it will not be stolen, and well, it’s good that they take it! Because, well it is useful for the doctor who is interested to really read it. But there should be enough for everyone …*”.

It is important to recognize that patients seek care in places other than the health post and health centers including from community healers and private providers. In La Unión, Zacapa an auxiliary nurse described the model’s emphasis on coordination between traditional healers and the MoH staff: *“There are people who seek community healers, because a lot of work was put in to the coordination with healers and people trust them a lot.”* Whereas in San Pablo, Sololá, a Tz’utujil-speaking patient explained through a translator that her mother had taken her to: *“a person who cures, in other words, who gives natural treatment …* and assisted a private clinic “… *that gives natural medicines and they take her blood pressure, and all of that. And they measure her sugar too.”*

### Human resources

Insufficient staffing along with frontline staff being assigned many tasks, makes it challenging for providers to complete everything that is expected of them: *“One of the needs has always been human resources, talking in general terms for Zacapa and health districts and health posts … in health centers there are a lot of activities and sometimes a nurse is assigned a lot of tasks and maybe they are within (his/her) competency range but because there are so many things, maybe, s/he is not able to do them.”* (Health Area-level staff, Zacapa).

A system-level problem is high staff turnover which has implications for sustaining processes of care and is linked to positions not being permanently budgeted: *“… the staff also has high turnover. Even if you train staff, after a year, it would be necessary to return and train them, something which is not a part of the culture to offer continuing education.”* (Central-level actor).

Actors at different levels pointed to a need for training and continuing education – on hypertension and specified some of the topics for which additional training would be beneficial: *“Health staff at the primary care level who are auxiliary nurses need to have the capacity to measure blood pressure adequately which requires training that is not difficult, but all health staff should have it.”* (Central-level actor).

Auxiliary nurses described the limited training that they received about hypertension when they were students, which underscores the importance of building in on-the job training and retraining: *“I think a half day. Not even, a half hour or an hour because these topics are covered very little. The topics that they spend the most time teaching are about pregnancy, birth … it is what they taught us the most.”* (Auxiliary nurse, San Pablo, Sololá) *“They taught us to take vital signs and also they taught us the normal blood pressure ranges... It is all that they taught us, the blood pressure ranges – the diastolic, systolic, so I can detect high blood pressure and normal blood pressure … I think that is the only training that I received about it.* (Auxiliary nurse, San Pablo, Sololá).

Providers expressed a desire to learn more about hypertension and shared thoughs about what they would like to learn. One frontline provider explained that they wanted to learn: *“… what you can do with a patient if their blood pressure does not go down, or treatments, or especially lifestyle approaches for health patients.”* (Auxiliary nurse, La Unión, Zacapa) Another provider referred to wanting to learn more about how to orient patients in disease management: *“I especially think it would be important for us to be prepared to provide patients the correct information, to be able to tell patients what they should do … and maybe … form a support group like with alcoholics where they share experiences...”* (Professional nurse, La Unión, Zacapa).

### Information systems

Limited capture and monitoring of hypertension in practice means that it is difficult to track key information such as how many patients in a unit have hypertension, and how many are controlled as described by an auxiliary nurse and central-level actor: *“In my case, I don’t know who the people are with hypertension. I know there are people, people have come here with this problem but I don’t think they are receiving follow-up. I don’t think so. Who knows how they are now.”* (Auxiliary nurse, San Pablo, Sololá).

*“If I go to a health care unit and ask: ‘How many of your hypertensive patients are controlled?’ they do not know, or ‘How many of these hypertensive patients were referred to the hospital because they did not achieve control?’ they do not know.”* (Central-level actor).

However, providers in La Unión, Zacapa who implement a servive delivery approach that does include care for patients with chronic conditions shared that they use lists to track patients identified with high blood pressure, diabetes and other chronic conditions: *“… each health post has a list of people with hypertension. So according to this I verify how many there are in each community and that way I can plan to go to the communities to visit them.”* (Professional nurse, La Unión, Zacapa).

Hypertension is undercounted on clinical forms but is evident in mortality data. A central-level actor described the disconnect between data gathered at the primary care level and cause of death statistics which shows an important gap that is at least in part due to not registering hypertension on clinical forms: “*If I have a patient with hypertension, I have to register it in SIGSA 3, the form for consults, but starting there, there is undercounting. One of the big problems with the information system is undercounting. Suppose that a patient comes in with pneumonia, but he also has hypertension, possibly in the SIGSA form hypertension will not be registered - just pneumonia will be … However, it shows up in the mortality. In 2016, I have a little graph that I carry around with me. For the primary causes of morbidity, hypertension is in 16*^*th*^ *place … But if you go to the primary causes of mortality, hypertension which was in 16*^*th*^ *place is up to 4*^*th*^ *place. So what happens with these patients? We do not detect them until they die.”*

### Medications and technologies

Hypertension medications are in short supply. Frontline healthcare providers shared that enalapril was the medication that was often (but not always) available at the health post level, of the three classes of medications for hypertension that should be available. An auxiliary nurse explained: *“In terms of treatment, what the service offers is enalapril, nothing more, because that is all that we have access to.”* (Auxiliary nurse, La Union, Zacapa) Two providers described the challenge of trying to treat patients with medicines other than enalapril. *“For now just enalapril. As I said, we have treated some patients by trying to … get samples, although sometimes it is a little concerning because we are able to get a good amount but then we run out and do not have any.”* (On-call doctor, San Pablo, Sololá). *“The only medication that the Health Area gives to us is enalapril, 20mg. So for people with hypertension that receive care here, it does not work for everyone … Some patients need other medicines and that is, well … the patients do not have money to buy them.”* (Health district staff, La Unión, Zacapa).

Patients do not always take their medicines or they take them intermittently. A patient explains his process for deciding when to get medicines: *“I don’t remember which doctor but yes, always when they check my blood pressure it is high … sometimes when my medicines run out I hold off on buying more or going to the health center. And then I start to get a headache. And that is when.. they tell me, they confirm there that my pressure is altered.”* (Patient, San Pablo, Sololá).

Patients also referred to taking traditional medicines in addition to or instead of pharmaceutical medications and providers, especially in La Unión, Zacapa, spoke about their coordination with local healers and their familiarity with traditional medicines. A professional nurse explained: “… *In the moment that we take someone’s blood pressure and detect that it is high, we also recommend traditional medicine and explain about nutrition, that they should not each much salt or food with a lot of condiments.*”

Lack of blood pressure monitors in some facilities. An auxiliary nurse in Zacapa explained that they have been working with their own blood pressure monitors: ***“****The monitors that we have are our own … At the beginning of the year when I began there were monitors, but here things deteriorate. So here what we have is our own.*

Community members are interested in learning how to use blood pressure monitors. Patients typically do not have blood pressure monitors and for the most part are not involved in monitoring their blood pressure, but expressed interest in doing so.

### Financing

Many respondents referred to the low level of public funding which is a problem for the public health system, and for the government budget more generally: *“It is not just in the Ministry of Health, but at the national level. The problem is that there is no budget. And that is due to the low tax collection in the country … the Ministry of Health does what it can with the limited budget that it has, which is less than 1.5% of the gross domestic product. In other words, they are actions that are palliative, right, just to cover the, the most urgent needs but to make a substantive change, that is not possible. And usually politicians do not understand - or do not want to understand - that. And so as long as that does not change, that the resources for the State are not more, the Ministry of Health and the whole government will have very limited functions.”* (Central-level actor).

Community members shared their experiences of not encountering medicines in public health facilities and having to buy medications, which is an economic hardship. *“I go running over, since I live near to the health center. I have had consults with the doctors … but sometimes they just give a prescription to you … sometimes they do not have (medications) and then you just get the prescription.”* (Patient, La Union) A community leader explained: *“One of the problems is that sometimes we do not have economic resources to buy certain medications. Sometimes we go to the health post and if there are not certain medications, well, we have to travel in to town, and if we do not have enough money, we can’t buy the medications.”* (Community leader, La Unión, Zacapa).

### Governance and leadership

A central-level actor explained the challenge of making progress in the area of chronic diseases due to limited prioritization: *“In terms of non-communicable diseases, the primary limitation is that authorities at high levels have never recognized the importance that they represent for public health. So, all policies and actions that the chronic disease program promotes have a lot of difficulty getting approval from the health minister and other government entities like the congress.”* (Central-level actor).

And among chronic diseases, hypertension is not as prioritized as diabetes. *“There is a little more follow up in patients with diabetes than among those with hypertension … I think it is because we are not promoting this follow-up which is different, for example, our Health Area has services like the clubs for diabetics … although sometimes they go hand-in-hand but we focus more on diabetes.”* (Health Area level staff, Sololá).

In both locations (Zacapa and Sololá), patients expressed interest in being more actively involved in their care and having their family members involved too. They stated an interest in learning more about hypertension and monitoring their blood pressure at home.

#### Cross-cutting issues affecting hypertension prevention and care

We identified four cross-cutting issues that were relevant to multiple building blocks: maternal and child health orientation of the health care system; emphasis on curative care vs. prevention; care-seeking and accessing resources in the community; and being accustomed to limited resources and shortages.

### Maternal and child health orientation of the health care system

The maternal and child health orientation of the health care system has de-emphasized services to address non-communicable diseases which has implications for what services are prioritized, what is covered in trainings, and what care patients expect to receive within health facilities. A central-level actor explained the relative prioritization of chronic disease programs as compared to maternal and child health and other priority programs: *“Chronic diseases have existed but have not been prioritized … Because the ones that appear (as priorities) are, for example, immuno-preventable diseases, diarrhea and pneumonia, and above all in population groups maternal and child health is going to be the priority.”* The orientation of the system toward maternal and child health and other related priorities leaves little time to train providers on chronic diseases, as described by this central-level actor: *“Basically, there is not training on chronic diseases … If you look at the list of all of the trainings that staff … receive in the year, they don’t go beyond nutritional aspects and sexual and reproductive health. That is how they spend their time, right, that’s what everybody talks about, but hypertension or its structural causes, that is not covered.”*

### Limited emphasis on public health as compared to curative care

A central-level actor refered to the curative orientation of the health system and implications of not providing preventive care: *“At the national level, as we are speaking, what always happens with health is that when people come to our services it is because they are already sick.”* Another central-level actor spoke to the budget for prevention being very small due to the emphasis on curative care and hospitals, as well as staff salaries: *“Much of the budget … is for human resources and the little that is left goes to curative care and in this part, to hospitals. And for prevention … the budget is very small.”* A family member of a patient with hypertension in La Unión, Zacapa referred to the perceived benefit of learning more about how to prevent disease: *“… if they told us what we should not consume. Because sometimes one does not know and maybe there are things that are causes and then knowing that, one would be more careful.”* If there were more of an investment in preventive care and education, the interest from family members and patients to learn more, could be leveraged.

### Care-seeking and accessing resources in the community

Patients and providers mentioned that care-seeking outside of MoH health posts and centers is common. Patients seek care and tratement from traditional healers, private providers, pharmacies and also seek advice from family members. It is important to recognize that this care-seeking within the community is part of the backdrop of care, and MOH providers may leverage resources within families and communities, especially to offset shortages in medication supply. In addition, patients often value traditional medicine and express a preference for it as was discussed in this focus group with patients in La Unión Zacapa: “*So the majority of people here we go to, and in fact, I will be honest, that I trust traditional remedies more than (pharmaceutical) medicines. I know that not all illnesses will be cured by traditional remedies but I trust them a lot.*”

### Being accustomed to limited resources and shortages

Providers, patients and community members commonly mentioned that they expect there will be medication shortages and insufficient staffing at health posts and centers. Central-level actors also referred to being used to limited resources and shortages and specifically referred to low levels of public funding, gaps in physical infrastructure, and not enough providers for all of the tasks that need to be completed. This influences how care is provided, patients’ care seeking behavior, and the expectation of what is possible with respect to hypertension control care.

Table 3 organizes primary needs identified during interviews and focus group discussions into short-term needs that may be addressed during the hypertension control study and long-term needs that will require investment beyond the scope of the hypertension control study. Key short-term needs included: making guidelines available, health care provider training on blood pressure management, standardization of clinical forms, increased capacity to make medications routinely available and enouraging patients to have a more active role in hypertension management. Key longer-term needs included:improving physical infrastructure, addressing staff turnover, developing measures to track hypertension care, increasing the overall public sector health budget and increasing an emphasis on prevention.

**Table 3:** Ways to address short- and long-term needs related to hypertension within Guatemala’s public primary care system

Health system building block	Short-term needs (that may be addressed by the project)	Long-term needs (requiring an investment beyond the scope of the project)
***Service delivery***	• Disseminate treatment guidelines for the detection and management of hypertension to all Ministry of Health providers at the primary care level • Develop a standardized approach to the collection and assessment of blood pressure indicators	• Improve coverage and physical infrastructure and access to medical supplies • Integrate prevention and health promotion into the approach to address hypertension
***Human resources***	• Offer in-service health care training on hypertension management to providers at all levels • Train providers on involving patients in blood pressure management	• Reduce staff turnover and increase professional/career advancement opportunities • Update the curriculum for auxiliary nurses to cover training in hypertension management
***Information systems***	• Standardize clinical forms and documentation of hypertension • Implement communication between patients and providers to share health data to improve management	• Develop hypertension & NCD health and service delivery measures that are feasible to track
***Medications and Technologies***	• Improve administrative capacity to request needed quantity of medications • Capture traditional health remedies that patients use to manage their blood pressure	• Ensure that all medications in the treatment guidelines are consistently available in health centers and posts • Increase laboratory capacity for diagnosis, treatment and management of NCDs • Increase the affordability of medications
***Financing***	• Increase the investment in preventive health vs. curative care • Estimate the cost of care for patients with hypertension	• Increase the Ministry of Health’s budget • Shift funding to match the epidemiologic profile of the country • Reduce high-out-of-pocket costs for patients
***Leadership and Governance***	• Increase community members’ participation in hypertension management • Empower patients with hypertension to seek treatment	• Establish intersectoral collaborations to address chronic conditions • Invest resources in regulation and health promotion • Develop a national strategic plan to cultivate high-level support for NCDs

Table 4 is a summary of the capacity at the health post level which was captured during visits to 124 health posts prior to implementing the intervention. Data is summarized for each of the 5 departments where the intervention is being implemented. The department of Huehuetenango is larger in size and population than the other four departments with health centers and posts serving a larger number of people; despite this larger population, the number of people 40 years of age and older per health post is lower in Huehuetenango (16%) than for two other departments (Baja Verapaz, 21%, and Chiquimula, 24%). The vast majority of health posts have all of the basic infrastructure (closed buildings with walls, space to store patient records, and a pharmacy). Huehuetenango and Chiquimula are the two departments which have a higher percentage of health posts with limited access, since the urban seat can only be reached by foot from 29% of the health posts in Huehuetenango and only by 4x4 vehicle from 27% of the health posts in Chiquimula. Health posts in Chiquimula, Huehuetenango and Sololá are staffed with more than 2 auxiliary nurses, on average as compared to Zacapa and Baja Verapaz that have a lower level of staffing. Baja Verapaz is the department with the greatest number of health posts (65%) that had experience with lists of patients with hypertension at baseline and also had the highest percentage of health posts with medications (enalapril - 91% and losartan – 57%). In contrast, Chiquimula had the lowest level of available medications. Hydrochlorothiazide, the first line medication in the MoH guidelines, was available in only 1 of the 124 health posts that were visited prior to implementation of the intervention. With the exception of Zacapa, blood pressure monitors were available in over 90% of the health posts in each of the other departments.

**Table 4:** Characteristics of infrastructure observed at health posts in five departments in Guatemala prior to implementing the hypertension control study

	Average across 5 departments	Zacapa	Chiquimula	Sololá	Huehuetenango	Baja Verapaz
**Population served**						
**Population served by health district (mean)**	32,981	27,040	32,310	31,004	40,620	33,931
**Population served by health post (mean)**	3239	2613	2451	3764	4675	2694
**Population > 40 years (mean/health post)**	540	418	583	612	529	560
**Average proportion of patients > 40 years/population (%)**	*17*	*16*	*24*	*16*	*11*	*21*
**ServiceDelivery**						
**Basic infrastructure at the health post level**	**(n = 97)**	**(n = 8)**	**(n = 26)**	**(n = 26)**	**(n = 41)**	**(n = 23)**
Closed building with walls (%)	*95*	*100*	*85*	*100*	*100*	*100*
Space to store patient records (%)	*97*	*100*	*85*	*100*	*98*	*91*
Pharmacy (%)	*97*	*100*	*85*	*100*	*98*	*100*
**Additional Infrastructure**						
Clinic (%)	99	*100*	*96*	*100*	*100*	*100*
Patient record file cabinet (%)	87	*100*	*81*	*69*	*90*	*96*
Space to put a patient file cabinet (%)	91	*100*	*85*	*96*	*85*	*91*
Space to have hypertension club meetings (%)	75	*63*	*62*	*100*	*85*	*65*
**Access to the health post (transport options from the municipal seat to the health post)**						
Only by walking (%)	6	*0*	*0*	*0*	*29*	*0*
Only in a 4x4 vehicle (%)	10	*0*	*27*	*8*	*5*	*9*
Only by boat (%)	1	*0*	*0*	*4*	*0*	*0*
Any type of vehicle (%)	84	*100*	*73*	*89*	*66*	*91*
Mean time (minutes)	33	32	26	35	37	37
**Human resources**						
# auxiliary nurses/health post (mean)	2	1.38	2.27	2.40	2.22	1.70
**Information systems**						
Health posts that have a list of people with hypertension (%)	*30*	*25*	*15*	*12*	*34*	*65*
**Medications andMedicalSupplies**						
**Medication type (available at the health post: yes/no)**						
Hydrochlorothiazide (%)	*1*	*0*	*4*	*0*	*0*	*0*
Enalapril (%)	*77*	*100*	*15*	*89*	*88*	*91*
Losartan (%)	*11*	*0*	*0*	*0*	*0*	*57*
**Medical supplies (available at the health post: yes/no)**						
Blood pressure cuff (%)	*85*	*50*	*96*	*96*	*90*	*91*
Stethoscope (%)	*93*	*100*	*89*	*96*	*93*	*96*
Adult weight scale (%)	*81*	*75*	*73*	*81*	*90*	*87*
Adult height rod (%)	*80*	*88*	*77*	*81*	*71*	*83*

## Discussion

The WHO building blocks framework provided a helpful way to structure and organize our data collection for this complex multi-level public health intervention research effort. Major system-level challenges we identified were a low level of public health funding, limited prioritization of hypertension, a gap in infrastructure, especially in rural areas, insufficient staffing and high staff turnover. Key opportunities we identified include: the strong interest in increasing the prioritization of hypertension and NCDs within the healthcare system, an eagerness among providers to learn more about hypertension, previous experience in some settings in identifying and monitoring patients with hypertension such as with the Inclusive Health Model [18, 19], and patients’ expressed interest in being more actively involved in their care.

A re-orientation of service delivery and the health information system toward indicator monitoring, use of registries, evidence-based proactive care, and emphasis on patient involvement in self-management would move the system toward the implementation of a Chronic Care Model approach proposed by the Pan American Health Organization [20].

The primary short-term needs within the public primary health care system identified during the needs assessment that fall within the scope of our hypertension control study were: limited availability of treatment guidelines, low level of training in hypertension prevention and care, lack of consistent reporting of hypertension in practice, and limited access to medications other than enalapril at the community level; enalapril was preferred since it is cheaper than hydrochlorothiazide. The need for detailed training and adequate medications and supplies have been identified in other settings as captured in a review of barriers and facilitators to the delivery of non-communicable disease care by non- physician health workers identified in low- and middle-income country settings [21].

Our multi-method needs assessment has a number of operational implications for our trial which is underway. As a team, we recognize the importance of closely monitoring the availability of medications within each of the health posts participating in the intervention. We have decided to capture this information on a monthly basis at each of the health posts and centers using a modified version of the data collection forms used during the implementation of this needs assessment. Due to the challenge of high staff turnover, we have identified the need to invest resources in the retraining of healthcare providers; in coordination with the service delivery unit of the MoH, we are ensuring that providers who are newly hired during the study period receive training on the hypertension control approach.

The longer-term needs for health system strengthening that fall outside of the scope of this project will be important to address in order to enable a sustainability infrastructure within the MoH to sustain and scale-up the intervention proven effective during the trial [15]. In a systematic review of quality improvement of cardiovascular disease care in LMICs, most studies focused on the patient/provider levels; for those focusing at the system level, the introduction of universal health coverage was found to improve hypertension and diabetes control [22] which is in line with the global communities’ prioritization for health within the sustainable development goals [23]. The Pan American Health Organization has recently gone a step further to emphasize prevention and the “causes of the causes” with a social determinants of health perspective [24].

.This study had a number of notable strengths. First, we thoroughly captured the perspectives of multiple stakeholders (MoH Health Area and District staff, doctors, nurses, auxiliary nurses, patients, family members, community members) in two different settings where the study is being implemented. Second, we conducted interviews at the central level which shed light on the national-level context. In addition to the qualitative data, we captured needs in frontline health units through visits to health posts and centers in each of the five departments where the intervention is being implemented. Lastly, our needs assessment has directly informed the current study that we are implementing and also presents an overview of longer-term needs. Those needs are related to the sustainability infrastructure that will enable the scale-up of this intervention if proven effective and will be central to reducing existing inequalities in cardiovascular health care [11].

Our study also had several limitations. The need to consider and capture data on the dynamic context [25] has become fully evident with the COVID-19 pandemic which began during the implementation of our study [26]. In the future, we recommend using a multi-level and multi-method form to routinely capture relevant contextual data applying the PRISM and building blocks frameworks. While our study was very thorough, the tradeoff is that the large amount of data that we gathered limited our ability to make full use of it in a timely manner. In the future and during our ongoing study, our team recognizes that it will be important to be more selective in the data that we capture in order to streamline the process and conduct rapid analysis [27] on specific topics of interest to inform timely decisions and understand changes in the context.

At the same time, our team continues to deepen our understanding of hypertension prevention, detection, and care through more pointed analyses of the data gathered during the needs assessment including an analysis of the explanatory models of hypertension as we recognize that participants refer to and understand the condition - and health more broadly – in different ways [28]. While our study was thorough, there are certain domains that are important to implementation science that we did not focus on in our assessment; in particular, we would benefit from capturing information related to the inner setting such as readiness for change of each of the districts that may influence the implementation and future scale-up of the intervention [29]. We plan to explore this and other aspects – including equity and sustainability – in an ongoing way during the study [30]. In applying the building blocks framework, we recognize the limitation of breaking the system apart in to individual blocks which are not independent of one another; in future needs assessments, we propose to adopt more of a systems thinking approach and are interested to implement stakeholder engaged methods such as participatory system dynamics modeling [31]. Furthermore, while a focus on the specific condition of hypertension allowed us to narrow our needs assessment, a syndemics perspective [32] coupled with systems thinking may enable a more public-health oriented view that is needed to break down silos to improve overall prevention and care of NCDs, and embrace their interaction with communicable diseases.

Our needs assessment is relevant for other LMIC settings that are working to expand NCDs service offerings in order to respond to changing epidemiological profiles. If our intervention is proven effective, we will conduct a similar needs assessment in neighboring countries prior to supporting a scale-out effort.

## Conclusions

This needs assessment provided essential insights that allowed our team to tailor the design of the hypertension control intervention approach and identify priority areas for ongoing and rapid qualitative and quantitative data capture and analysis throughout the trial. In addition, our multi-methods needs assessment has enabled our team to identify what we have the potential to resolve in the short-term and what lies outside of the scope of the project and requires longer-term, system strengthening efforts. Our study contributes both to the WHO health systems building blocks approach and to the field of implementation science as it relates to understanding how to capture and analyze multi-level health system contextual data for chronic disease service delivery programs in LMICs. Our findings are relevant to and will aid initiatives aimed at improving care for hypertension control and additional NCDs including diabetes, chronic kidney disease, and cancer within Guatemala and in other similar LMIC settings.

## Supplementary Information

**Additional file 1: Supplementary file 1.** Interview Guide.

**Additional file 2: Supplementary file 2.** Coding Tree.

## Data Availability

Data from this study are available from the corresponding author upon reasonable request.

## Abbreviations

LMICs: Low- and middle-income countries
NCDs: Non-communicable diseases
WHO: World Health Organization
HyTREC/TREIN: Hypertension Outcomes for T4 Research within Lower Middle-Income Countries/T4 Translation Research Capacity Building Initiative in Low Income Countries
NHLBI: National Health Lung and Blood Institute
RE-AIM: Reach, Effectiveness, Adoption, Implementation, Maintenance
PRISM: The Practical, Robust and Sustainability Model
FGDs: Focus group discussions
SIGSA: Sistema de Información Gerencial de Salud (in Spanish; English translation: Health Management Information System)
PAHO: Pan American Health Organization
