# Supplementary material for: Hypertension in Guatemala’s Public Primary Care System: A Needs Assessment Using the Health System Building Blocks Framework
Source: BMC Health Serv Res. 2021 Sep 3;21:908. doi: 10.1186/s12913-021-06889-0 (PMC8414027; doi:10.1186/s12913-021-06889-0)
Supplement: Supplementary file 1 — Additional file 1: Supplementary file 1. Interview Guide. [file 12913_2021_6889_MOESM1_ESM.docx]

**Appendix 1. Health system needs assessment interview and focus group discussion guides**

For the needs assessment, we will ask you some questions about the different components of the health care system, related specifically to hypertension. The components are: 1) provision of services; 2) health care providers; 3) information systems; 4) access to medications; 5) financing; and 6) leadership/governance.

**(1) For key informants (Central-level, Ministry of Health):**

- First, I would like to ask you some questions about your role and background: What is your job title? For how long have you been in this role? What is your professional field?
- To continue, I would like to ask about needs that you have observed within the health system: What are the needs/limitations? What do you consider the needs to be with respect to your unit? With respect to: the provision of services, health workforce, information systems, access to essential medicines, financing, and leadership and governance? Do you think that they may be overcome? If so, how?
- Focusing on hypertension…
  - What is the vision that you have of patients with hypertension at your institution? How would you describe a patient with hypertension?
- In general, which do you think is the part of the health system that most needs strengthening to provide quality health care services to people with hypertension?
- Do you lead/engage in activities to promote the prevention of high blood pressure? Which activities do you lead/engage in and who do they target? What else would you like to do to promote the prevention of high blood pressure in the population?
- As for the provision of services:
  - Upon detecting blood pressure, what are the next steps for the patient?
  - What responsibility does the patient have to monitor their blood pressure at home? What tools does he/she have to follow up on their blood pressure (i.e. nutritional recommendations, a sheet to record their blood pressure readings, a blood pressure monitor)?
- As for the health care personnel:
  - What training do the physicians/nurses/auxiliary nurses receive regarding the prevention and control of hypertension?
  - Do you believe that health care worker training is adequate regarding the prevention and control of hypertension?
  - What additional training would help them?
- As for the information system:
  - At the national level, how do you estimate the incidence/prevalence of hypertension in the population?
  - How many people are reported to have hypertension? How is this information obtained?
  - Where are hypertension related deaths reported/registered? Do you believe that most deaths are registered? How could the system for capturing deaths related to hypertension be improved?
  - In which forms is hypertension registered? Is there space to include blood pressure measurements? How many measurements? How many cells are there available to record diagnoses? How many cells are available to record medications? Where is the patient’s follow up plan registered?
  - How do you believe that hypertension registration could be improved in SIGSA?
- As for access to essential medications, which medicines does the MSPAS purchase?
- How does the distribution system work?
- How is it defined which quantities to purchase?
- Medications:
  - Hydrochlorotiazide, Ramipril, Cilazapril, Enalapril, Captopril, Irbesartan, Losartan
  - Other medications?
- As for funding:
  - How much money per year is budgeted for the prevention of hypertension?
  - How much (per capita) is devoted to hypertensive patients?
  - What percentage of funds is devoted to the prevention and control of hypertension in primary care?
  - How much do you believe is required to offer quality health care services?
- As for leadership/governance:
  - Do you believe that the prevention and control of hypertension is a priority in the health care system in Guatemala? Would you categorize the prevention and control of hypertension as a priority? If you do think it is a priority, how would you improve prevention and control of hypertension? In your institution, are there activities to promote the prevention of hypertension? What activities are they and who are they directed toward? What else would you like to see to promote hypertension prevention in the population?

Is there anything that you would like to ask me or a comment that you would like to add? Is there another topic that you would have liked to talk about that we did not cover?

We thank you very much for your participation.

**(2) For administrators (area/district directors):**

- What is your current role?
- What do you consider to be the priority needs that you observe within your district? How do you think they may be addressed?
- As for the provision of services:
  - Upon detecting blood pressure, what are the next steps for the patient?
  - What responsibility does the patient have to monitor their blood pressure at home? What tools does he/she have to follow up on their blood pressure (i.e. nutritional recommendations, a sheet to record their blood pressure readings, a blood pressure monitor)? Do you promote patient-level prevention activities?
- As for the health care personnel:
  - Have you held a specific training on the prevention and control of hypertension?
  - Do you believe that health care worker training is adequate regarding the prevention and control of hypertension?
  - What additional training would help them?
- As for the information system:
  - How many people are reported to have hypertension? How is this information reported?
  - In which forms is hypertension registered? Is there space to include blood pressure measurements? How many measurements? How many cells are there available to record diagnoses? How many cells are available to record medications? Where is the patient’s follow up plan registered? How do you believe that hypertension registration could be improved?
  - Where are hypertension related deaths reported/registered? Do you believe that most deaths are registered? How could the system for capturing deaths related to hypertension be improved?
- As for access to essential medications, which medicines does the MSPAS purchase? How does the distribution system work? How is it defined which quantities to purchase?
- Medications: Hydrochlorotiazide, Ramipril, Cilazapril, Enalapril, Captopril, Irbesartan, Losartan. Other medications?
- As for funding:
  - How much money per year is budgeted for hypertension?
  - How much (per capita) is devoted to hypertensive patients?
  - What percentage of funds is devoted to the prevention and control of hypertension in primary care?
  - How much do you believe is required to offer quality health care services?
- As for leadership/governance:
  - Do you believe that the prevention and control of hypertension is a priority in the health care system in Guatemala? In your district? How would you improve prevention and control of hypertension?

Is there anything that you would like to ask me or a comment that you would like to add? Is there another topic that you would have liked to talk about that we did not cover?

We thank you very much for your participation.

**(3) For health workers (physicians, nurses, auxiliary nurses):**

- What is your current role and how long have you been in it?
- Describe the patients that you serve in your health center/post.
- What are the primary difficulties that patients with blood pressure experience? Probes: Financial, follow up, medications, other.
- Human resources: What training did you receive on the prevention and control of hypertension? Aside from your medical/nursing training, have you received specific training (workshop/refresher course) on the prevention and control of hypertension? What else would you like to know about the prevention and control of hypertension?
- Service delivery: Once high blood pressure is detected, what are the next steps to take with the patient? Who on the team is in charge of taking care of hypertensive patients? Do you believe the patient has the responsibility of monitoring their blood pressure at home? Which tools does he/she have to monitor his/her blood pressure at home?
- Information system: In the area you are responsible for, how many people are identified as being hypertensive? In which forms is hypertension registered? Is there space to include blood pressure measurements? How many measurements? How many cells are there available to record diagnoses? How many cells are available to record medications? Where is the patient’s follow up plan registered?
- As for access to essential medications, which medicines for hypertension are available in your district/sector?
- Medications: Hydrochlorotiazide, Ramipril, Cilazapril, Enalapril, Captopril, Irbesartan, Losartan. Other medications?

**(4) For patients:**

- Let’s begin with some background about you. What activities do you dedícate your time to? How do you consider your health, overall?

Focusing on blood pressure:

- Are you currently experiencing blood pressure issues?
- How did you discover you were hypertensive? How old were you? When did you start taking medications?
- Does anybody else in your family have high blood pressure?
- Service delivery and human resources: How often do you receive care for your high blood pressure? With whom? Do you go to the health care center/post? Do you receive care anywhere else?
- Medications and cost: How many medications do you take for your high blood pressure? Where do you get the medications from? From the health center/post? From a private pharmacy? How much do they cost? Do you have any difficulty getting them? Do you take additional treatments other than pharmaceutical medications?
- Self-management (governance and information): How often do you monitor your blood pressure? Do you know what your usual blood pressure reading is?
- Since you have high blood pressure, have you changed your diet or physical activity levels? Did you receive any education about changes in your diet or physical activity levels?

Additional interviews were conducted with family members, community leaders, and providers who do not work within the Ministry of Health.

**Implementing a Multicomponent Intervention to Improve Hypertension Control in Central America, stage 1: Guatemala**

**Focus group discussion guides - for health care workers and patients**

Questions for health care workers:

- What is your role within the Ministry of Health? And within the community?
- Describe what you do when you find a patient who has high blood pressure.
- How often do you follow-up at the beginning? What does the follow up consist of? How do you coordinate the work to support patients with hypertension?
- Which difficulties have you encountered when applying the recommended standard of care to control hypertension?
- Do you consider that patients understand what you convey/the treatment that you prescribe to them?
- What advice do you give patients to achieve hypertension control?
- What are the biggest challenges/obstacles that patients mention that they face in trying to follow your indications?
- What do you think helps patients achieve hypertension control?
- What training would you like to receive regarding hypertension control?
- Ask about feasibility of proposed hypertension control activities.

Questions for patients:

- When you became aware that you had high blood pressure, what type of follow up did the doctor, nurse or auxiliary nurse do? How often do you have clinic appointments now?
- What advice do you receive during your appointment? Regarding the advice you have received, what has been most helpful? What else would be helpful?
- What are the biggest challenges/obstacles you face in complying with the received indications?
- What helps you to control your hypertension?
- Ask about feasibility of proposed hypertension control activities.
