# Supplementary figures and images for: Hypertension in Guatemala’s Public Primary Care System: A Needs Assessment Using the Health System Building Blocks Framework
Source: BMC Health Serv Res. 2021 Sep 3;21:908. doi: 10.1186/s12913-021-06889-0 (PMC8414027; doi:10.1186/s12913-021-06889-0)

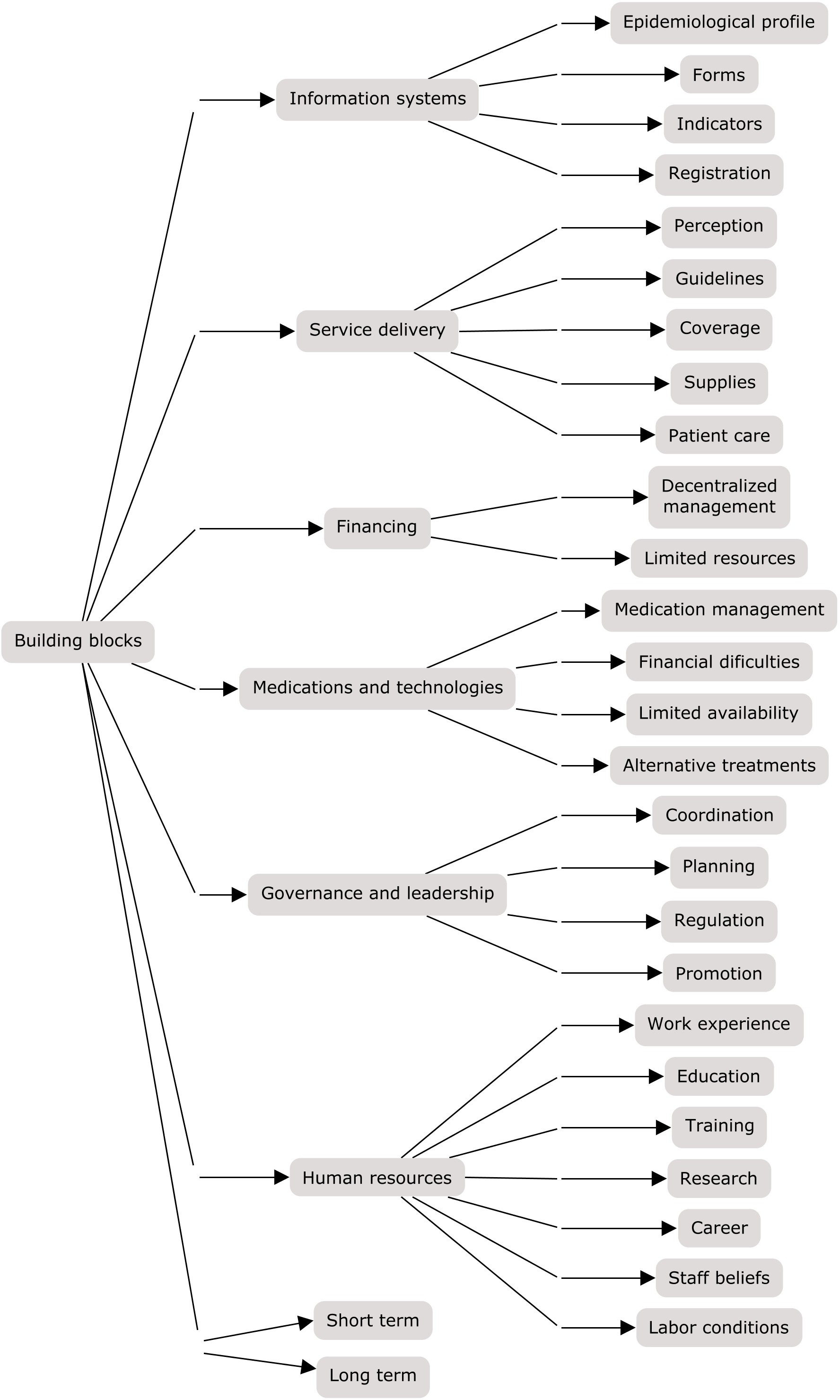

Supplement: Supplementary file 2 — Additional file 2: Supplementary file 2. Coding Tree. [file 12913_2021_6889_MOESM2_ESM.jpg]
